# Development and validation of a risk score to predict the frequent emergency house calls among older people who receive regular home visits

**DOI:** 10.1186/s12875-022-01742-7

**Published:** 2022-05-26

**Authors:** Yu Sun, Masao Iwagami, Nobuo Sakata, Tomoko Ito, Ryota Inokuchi, Kazuaki Uda, Shota Hamada, Miho Ishimaru, Jun Komiyama, Naoaki Kuroda, Satoru Yoshie, Tatsuro Ishizaki, Katsuya Iijima, Nanako Tamiya

**Affiliations:** 1 Graduate School of Comprehensive Human Sciences, University of Tsukuba, Ibaraki, Japan; 2 Department of Health Services Research, Faculty of Medicine, University of Tsukuba, Ibaraki, Japan; 3 Health Services Research and Development Center, University of Tsukuba, Ibaraki, Japan; 4 Institutes of Medicine, University of Tsukuba, Building 861, 1-1-1 Tenno-dai, Tsukuba, Ibaraki, Japan; 5 Research Department, Institute for Health Economics and Policy, Association for Health Economics Research and Social Insurance and Welfare, Tokyo, Japan; 6 Department of Home Care Medicine, Graduate School of Medicine, University of Tokyo, Tokyo, Japan; 7 Health Department, Tsukuba City, Ibaraki Japan; 8 Community Clinic Tsukuba, Ibaraki, Japan; 9 Institute of Gerontology, University of Tokyo, Tokyo, Japan; 10 Institute for Future Initiatives, University of Tokyo, Tokyo, Japan; 11 Department of Health Policy and Management, School of Medicine, Keio University, Tokyo, Japan; 12 Human Care Research Team, Tokyo Metropolitan Institute of Gerontology, Tokyo, Japan; 13 Department of Community and Public Health Nursing, Faculty of Medicine, University of Tsukuba, Ibaraki, Japan; 14 Department of Oral Health Promotion, Graduate School of Medical and Dental Science, Tokyo Medical and Dental University, Tokyo, Japan

**Keywords:** Home healthcare services, Risk score, Emergency house calls, High-risk patients

## Abstract

**Background:**

The demand for home healthcare is increasing in Japan, and a 24-hour on-call system could be a burden for primary care physicians. Identifying high-risk patients who need frequent emergency house calls could help physicians prepare and allocate medical resources. The aim of the present study was to develop a risk score to predict the frequent emergency house calls in patients who receive regular home visits.

**Methods:**

We conducted a retrospective cohort study with linked medical and long-term care claims data from two Japanese cities. Participants were ≥ 65 years of age and had newly started regular home visits between July 2014 and March 2018 in Tsukuba city and between July 2012 and March 2017 in Kashiwa city. We followed up with patients a year after they began the regular home visits or until the month following the end of the regular home visits if this was completed within 1 year. We calculated the average number of emergency house calls per month by dividing the total number of emergency house calls by the number of months that each person received regular home visits (1–13 months). The primary outcome was the “frequent” emergency house calls, defined as its use once per month or more, on average, during the observation period. We used the least absolute shrinkage and selection operator (LASSO) logistic regression with 10-fold cross-validation to build the model from 19 candidate variables. The predictive performance was assessed with the area under the curve (AUC).

**Results:**

Among 4888 eligible patients, frequent emergency house calls were observed in 13.0% of participants (634/4888). The risk score included three variables with the following point assignments: home oxygen therapy (3 points); long-term care need level 4–5 (1 point); cancer (4 points). While the AUC of a model that included all candidate variables was 0.734, the AUC of the 3-risk score model was 0.707, suggesting good discrimination.

**Conclusions:**

This easy-to-use risk score would be useful for assessing high-risk patients and would allow the burden on primary care physicians to be reduced through measures such as clustering high-risk patients in well-equipped medical facilities.

**Supplementary Information:**

The online version contains supplementary material available at 10.1186/s12875-022-01742-7.

## Introduction

In recent years, the organization of primary healthcare after office hours has changed in many countries. There are new models for after-hours care, such as large-scale general practice cooperatives, primary care centers integrated into hospital emergency departments, or telephone triage and consultation services [1]. These changes are partly due to primary care physicians’ reluctance to commit to being on-call 24-hour a day and 7 days a week because of the workload burden, increasing patients’ demand for after-hours care, and regional shortages of primary care physicians [2, 3].

In Japan, where the population is aging the fastest in the world [4], the demand for home healthcare has also increased due to the aging population and the government-sponsored shift of care from the hospital to the community [5]. All citizens in Japan have medical care coverage under a universal health insurance system, which consists of occupational insurance for salaried workers (employees), National Health Insurance for self-employed and retirees under 75 years of age, and Late-stage medical care system for the all elderly aged 75 and over [6, 7]. Japan also started a mandatory long-term care insurance system in 2000, distinct from the national medical insurance system [8]. Under the statutory long-term care insurance system, older people who need living assistance can receive care services based on the seven levels of the certificate of need for long-term care: Support 1 (lowest disability) to 2 and Care 1 to 5 (highest disability) [9]. Long-term care need level is a nationally standardized certification that is assessed based on a person’s physical and cognitive functioning [10]. All Japanese citizens who are ≥65 and individuals 40–64 years whose need of care is derived from aging-related diseases, such as stroke, cancer, and rheumatoid arthritis, are eligible for these benefits.

Home healthcare in Japan entails physicians making regular home visits to diagnose and monitor medical conditions, as well as prescribe medications. To be enrolled in physician-led home healthcare, patients apply by themselves or the primary care physician identifies patients who require home healthcare, under the condition that the patients cannot get to an outpatient clinic. In addition, they must reside within roughly 16 km of the hospital or clinic that provides these services. Physicians are required to provide regular home visits once or twice per month depending on the patients’ medical needs. Additionally, patients who receive physicians’ home visits often use the nursing care visits and home help services offered by a variety of care facilities [11].

To promote home healthcare, especially for emergency house calls and end-of-life care, the Ministry of Health, Labour, and Welfare introduced home care support clinics and hospitals (HCSCs) in 2006, with home care support functions available 24-hour a day until the patient dies [12]. HCSCs have a system that enables 24-hour emergency house call at the patient’s request. However, previous research has shown that more than 70% of physicians in HCSCs feel burdened by the 24-hour on-call coverage mandated for HCSCs [13]. To enhance home healthcare, it is essential to identify a high-risk population with frequent emergency house calls, and take measures to reduce physical and psychological burdens for primary care physicians.

Studies have shown that the common reasons for emergency house calls are fever, end-of-life care, dyspnea, and cough among patients who receive regular home visits in Japan [14, 15]. However, these studies focused on the chief complaint and did not consider factors of the patient’s condition such as comorbidities or medical procedures performed in the home care setting. In addition, they were single- or few-center studies, which limits their generalizability. To take measures to relieve the burden on primary care physicians, it is necessary to assess the risk of patients with frequent rates of emergency house calls. However, to date, no study has developed risk prediction models for the frequent emergency house calls.

Therefore, we developed and validated a risk score that includes comorbidities and medical interventions in home healthcare to predict frequent emergency house calls among older people who receive regular home visits.

## Methods

### Study design and data source

We conducted a retrospective cohort study. We obtained linked data on medical and long-term care insurance claims from the municipal governments of two cities (Tsukuba city, Ibaraki Prefecture, and Kashiwa city, Chiba Prefecture) in Japan. As both cities are suburbs in the Tokyo metropolitan area, we combined their data.

Medical claims data included data from individuals with National Health Insurance and Late-stage medical care system for the elderly for individual prefectures, while data from individuals with other health insurance credentials (e.g., insurance for corporate employees) were not included [6, 7]. Generally, the National Health Insurance covered 74% of the population in 2016 [16], aged 65–74, and the Late-stage medical care system covers the entire population, aged 75 and over. Medical insurance claims records included covered diagnoses, medical procedure information, and prescription information on a monthly basis. The recorded diagnoses were based on the original Japanese disease codes linked to the International Classification of Diseases 10th Revision (ICD-10) codes [17]. Long-term care insurance claims data contains information on the care need levels and services used for all residents receiving long-term care services.

The linkage between medical and long-term claims data was made in each municipal government using personally identifiable information. In the data we received, anonymized ID numbers were assigned to individuals in both medical and long-term care insurance claim datasets.

### Study population

**Fig. 1:**
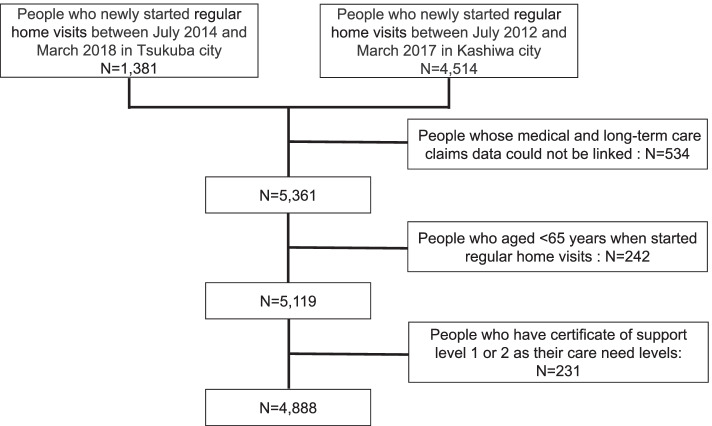
Flow chart of study participant selection

Individuals who had newly started availing regular home visits between July 2014 and March 2018 in Tsukuba city and between July 2012 and March 2017 in Kashiwa city were included (*n* = 5895). Individuals who did not receive regular home visits between April and June 2014 in Tsukuba city and between April and June 2012 in Kashiwa city were considered newly enrolled. First, we excluded people whose medical and long-term care claims data could not be linked (*n* = 534). Next, we excluded people who were < 65 years when they started regular home visits (*n* = 242). The age of 65 years was chosen as the lower limit because (i) all people ≥65 years are eligible for long-term care insurance benefits, (ii) the vast majority (over 95%) of regular home visits are conducted for this age group [18]. We then excluded those who had a certificate of support level 1 or 2 (*n* = 231). Long-term care need levels correlate well with the Barthel Index, an internationally accepted indicator for activities of daily living (ADL) [9]. While almost all the people with care need level 5 have a Barthel Index score of 0–40, most with support levels 1 or 2 had a Barthel Index score of ≥60 [9], which is the cut-off point for difficulty in performing basic ADL and dependence on the care of others [19]. Since home visits are generally performed for patients who are disabled and cannot visit a clinic or hospital, we excluded such people. Thus, a final sample of 4888 individuals was evaluated (Fig. 1).

### Outcome variable

The primary outcome of the present study was the “frequent” emergency house calls during the period of regular home visits. This was defined as the use once per month or more (on average) during the observation period. We followed up with patients 1 year after the start of the regular home visit or until the month following the end of the regular home visit if this was completed within 1 year. During the period, the total number of emergency house calls was determined using medical insurance records. We calculated the average number of emergency house calls per month by dividing the total number of emergency house calls by the number of months that each person received regular home visits (1–13 months).

### Predictor variable

For each patient, we identified variables potentially associated with the frequent emergency house calls, including age (categorized as 65–74, 75–84, 85–94, or ≥ 95 years); gender; medical procedures performed in home medical care including self-injection, central venous nutrition, enteral nutrition, home oxygen therapy [14], use of ventilator/tracheostomy performed, and urinary self-catheterization; long-term care need levels [14] classified as care need level 1, 2–3, and 4–5; medical diagnosis at the start of the regular home visit, including cerebrovascular diseases, cardiac diseases, lower respiratory tract diseases, joint diseases, dementia, Parkinson’s disease, diabetes, visual of hearing impairment, fractures, and cancer. Medical interventions performed in the month in which the regular home visit began were identified from medical insurance claims records. In contrast, the long-term care need levels were determined at the time of the most recent use of long-term care insurance services within 3 months of the start of the regular home visit. We identified medical diagnoses from medical insurance claims data during the 3 months before the start of the regular home visit. Medical diagnoses were categorized based on ICD-10 codes related to diseases associated with the initiation of long-term care in the Comprehensive Survey of Living Conditions in Japan [20] (Supplementary Appendix 1). The “suspected” diagnosis codes were excluded from the datasets.

### Statistical analysis

First, we compared those with frequent emergency house calls and the others by using chi-square tests or Fisher’s exact test when the expected frequency was less than 5. Then we performed multivariable logistic regression analysis with all candidate variables included.

To create the most efficient and easy-to-use risk score in actual clinical practice, we used the least absolute shrinkage and selector operation (LASSO) logistic regression, with 10-fold cross-validation and the largest lambda at which the mean-squared error (MSE) was within one standard error of the minimal MSE [21]. LASSO is an extended standard regression model, developed as a parsimonious prediction model by selecting important predictors [22]. The model resulting from LASSO is known to have better predictive model selection performance and predictor identification than classical regression methods [23]. A scoring system was derived by multiplying each beta coefficient from the LASSO logistic regression by 4 and rounding them to the nearest whole number [24]. The integer values of all applicable variables were then summed up to determine a total score for each patient. In the assessment of the discrimination ability of the prediction model, the receiver operating characteristic (ROC) curve for the risk score was drawn, and the area under the curve (AUC) was compared with the model in which all candidate variables were included. Calibration was assessed graphically by plotting the average predicted probabilities against the observed probabilities corresponding to the quintiles of predicted probabilities.

As a post hoc analysis, as we suspected that the discrimination ability of the prediction model varies with age, we compared the characteristics between the different age group (65–84 and over 85 years) using chi-square tests or Fisher’s exact for categorical variables and Mann-Whitney U tests for continuous variables. Thereafter, we assessed AUCs of the 3-factor risk score for the 65–84 and over 85 age groups, separately.

All analyses were conducted using STATA version 15 (Stata Corp., Texas, USA). Statistical significance was set at *P* < 0.05.

The development and validation of this risk model followed the Transparent Reporting of a Multivariable Prediction Model for Individual Prognosis or Diagnosis (TRIPOD) statement [25].

## Results

Clinical characteristics of the entire sample are summarized in Table 1. The mean age was 84.1 (standard deviation 7.4) years, and 40.3% of participants were male. In the first year after the start of the regular home visit or by the month after the end of the regular home visit, 13.0% (634/4888) had an emergency house call once a month or more, on average. The distributions of the average number of emergency house calls per month is shown in Supplementary Appendix 2. It showed right-skewed distributions, with 0 accounting for approximately 50%.

**Table 1:** Sample characteristics

	*N* = 4,888
	n (%)
**Mean age, years (SD)**	84.1 (7.4)
**Age category (years)**	
65−74	505 (10.3)
75−84	1895 (38.8)
85-94	2131 (43.6)
≥ 95	357 (7.3)
**Gender: male**	1972 (40.3)
**Medical procedure at home**	
Self-injection	99 (2.0)
Central venous nutrition	64 (1.3)
Enteral nutrition	14 (0.3)
Home oxygen therapy	292 (6.0)
Use of ventilator/ tracheostomy performed	30 (0.6)
Urinary self- catheterization	18 (0.4)
**Long-term care need levels**	
Care need level 1	850 (17.4)
Care need levels 2–3	2169 (44.4)
Care need levels 4–5	1869 (38.2)
**Medical diagnosis at the start of the regular home visit**	
Cerebrovascular diseases	1953 (40.0)
Cardiac disease	2783 (56.9)
Lower respiratory tract disease	2240 (45.8)
Joint diseases	2978 (60.9)
Dementia	2111 (43.2)
Parkinson’s disease	335 (6.9)
Diabetes	1615 (33.0)
Vision or hearing impairment	342 (7.0)
Fractures	892 (18.3)
Cancer	1404 (28.7)
**Month of receiving regular home visits: median (IQR)**	7 (2–12)
**Frequent emergency house calls**^a^	634 (13.0)

*Abbreviations*: *SD* Standard deviation, *IQR* Inter quartile range

^a^Emergency house calls once per month or more, on average, during each observation period

The characteristics associated with frequent emergency house calls in the univariable analysis (chi-squared or Fisher’s exact tests) and multivariable analysis are shown in Table 2. In the univariable analysis, patients in the group that made frequent emergency house calls tended to be 65–74 and ≥ 95 years old, male, more likely to be receiving central venous nutrition or home oxygen therapy, and had a higher long-term care need level. Regarding patients’ diseases, lower respiratory diseases and cancer were greater in the group with frequent emergency house calls, whereas those with cerebrovascular diseases, dementia, and fractures were less frequent. In the multivariable logistic regression analysis, home oxygen therapy, care need level 2–5 (compared with care need level 1), and cancer showed positive associations with frequent emergency house calls, whereas cerebrovascular diseases and dementia showed negative associations.

**Table 2:** Univariate and multivariable analysis of variables associated with frequent emergency house calls

	Univariable analysis			Multivariable logistic regression		LASSO logistic regression	Point score
	Non-frequent emergency house calls group (*n*=4,254)	Frequent emergency house calls^a^ group (*n*=634)	*P* value	OR (95%CI)	*P* value	β coefficient	
	n (%)	n (%)					
**Age category (years)**							
65−74	409 (9.6)	96 (15.1)	< 0.001	Reference	-	-	-
75−84	1654 (38.9)	241 (38.0)		0.85 (0.64–1.13)	0.264	-	-
85−94	1896 (44.6)	235 (37.1)		0.93 (0.69–1.25)	0.625	-	-
≥ 95	295 (6.9)	62 (9.8)		1.69 (1.14–2.51)	0.009	-	-
**Gender: male (vs. female)**	1649 (38.8)	323 (51.0)	< 0.001	1.25 (1.04–1.51)	0.020	-	-
**Medical procedure at home**						-	-
Self-injection	87 (2.1)	12 (1.9)	0.799	0.95 (0.50–1.80)	0.872	-	-
Central venous nutrition	46 (1.1)	18 (2.8)	<0.001	1.44 (0.81–2.58)	0.217	-	-
Enteral nutrition	10 (0.2)	4 (0.6)	0.097	1.81 (0.54–6.08)	0.339	-	-
Home oxygen therapy	192 (4.5)	100 (15.8)	< 0.001	2.81 (2.11–3.74)	<0.001	0.71	3
Use of ventilator/ tracheostomy performed	29 (0.7)	1 (0.2)	0.168	0.15 (0.02–1.16)	0.070	-	-
Urinary self- catheterization	15 (0.4)	3 (0.5)	0.721	1.69 (0.46–6.16)	0.428	-	-
**Long-term care need levels**			<0.001			-	-
Care need level 1	807 (19.0)	43 (6.8)		Reference		-	
Care need levels 2–3	1900 (44.7)	269 (42.4)		2.06 (1.46–2.89)	<0.001	-	
Care need levels 4–5	1547 (36.4)	322 (50.8)		3.23 (2.30–4.54)	<0.001	0.22	1
**Medical diagnosis at the start of the regular home visit**							
Cerebrovascular diseases	1742 (41.0)	211 (33.3)	<0.001	0.82 (0.68–0.99)	0.037	-	-
Cardiac disease	2425 (57.0)	358 (56.5)	0.800	0.92 (0.76–1.11)	0.382	-	-
Lower respiratory tract disease	1882 (44.2)	358 (56.5)	<0.001	1.17 (0.97–1.41)	0.096	-	-
Joint diseases	2588 (60.8)	390 (61.5)	0.744	0.89 (0.74–1.07)	0.220	-	-
Dementia	1920 (45.1)	191 (30.1)	<0.001	0.79 (0.65–0.97)	0.023	-	-
Parkinson’s disease	303 (7.1)	32 (5.1)	0.054	0.93 (0.63–1.38)	0.730	-	-
Diabetes	1390 (32.7)	225 (35.5)	0.160	1.05 (0.87–1.27)	0.617	-	-
Vision or hearing impairment	309 (7.3)	33 (5.2)	0.058	0.71 (0.48–1.04)	0.078	-	-
Fractures	802 (18.9)	90 (14.2)	0.005	0.86 (0.67–1.10)	0.233	-	-
Cancer	1056 (24.8)	348 (54.9)	< 0.001	2.97 (2.45–3.60)	<0.001	0.89	4

*Abbreviations*: *OR* Odds ratio, *CI* Confidence interval, *LASSO* Least absolute shrinkage and selection operator

^a^Emergency house calls once per month or more, on average, during each observation period

**Fig. 2:**
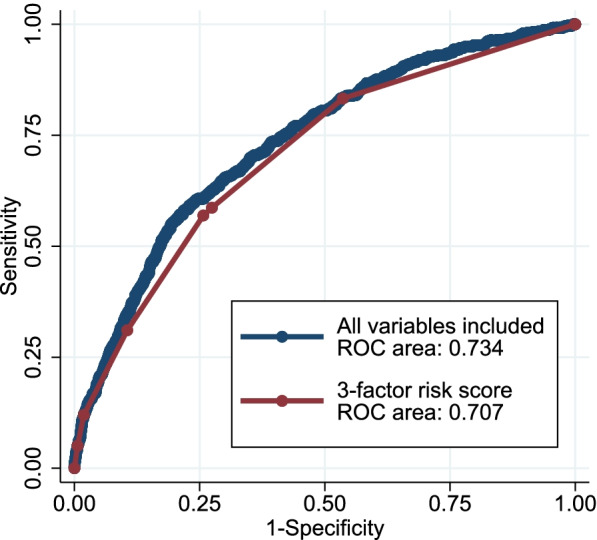
Receiver operating characteristic (ROC) curves and area under the curve (AUC) for the risk score

**Fig. 3:**
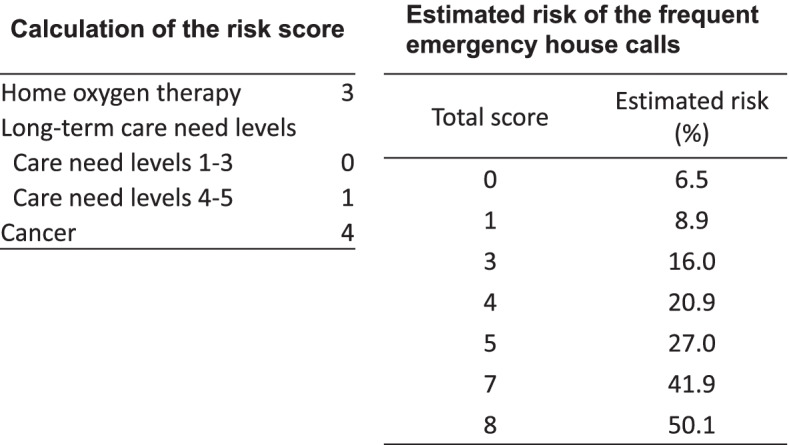
Calculation of scores and the corresponding estimated probabilities of the frequent emergency house calls

**Fig. 4:**
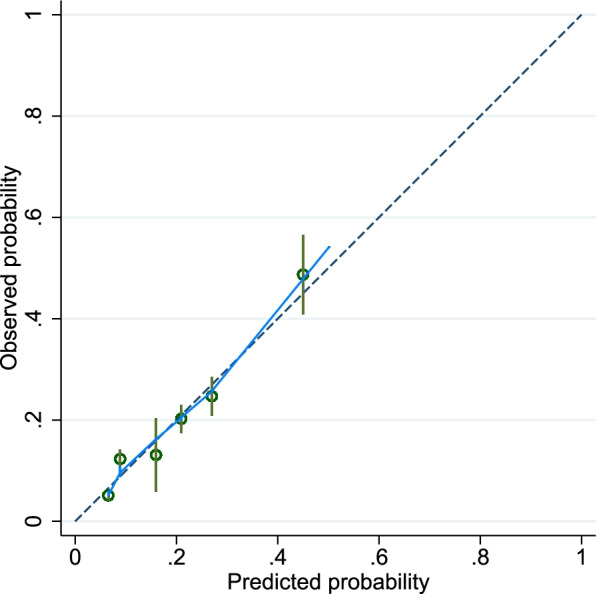
Calibration plot for predicting the frequent emergency house calls. The solid curve is the Loess-estimated calibration curve

Of the 19 candidate predictors included in the LASSO logistic regression, three were found to be significant predictors of frequent emergency house calls: home oxygen therapy, care need level 4–5, and cancer. The result of the beta coefficient and the created score are summarized in Table 2. The distribution of the total score is shown in Supplementary Appendix 3. The ROC curve and the AUC for the risk score are shown in Fig. 2. Compared with the model of all candidate variables (AUC; 0.734), the predictive ability of the 3-factor risk score (AUC; 0.707) was only slightly lower, which indicates a good discriminatory ability. The calculation of the score and the estimated probability of frequent emergency house calls are shown in Fig. 3. Figure 4 shows the calibration of the prediction model. The plotted points are relatively close to the 45° line, demonstrating good calibration over the whole range of the predictions.

In our post hoc analysis, the 65–84 age group had a higher proportion of males and more medical procedures done at home. Regarding medical diagnoses at the start of the regular home visit, Parkinson’s disease, diabetes, and cancer were more prevalent in the 65–84 age group, whereas cardiac disease, joint diseases, dementia, and fractures were more common in the over 85 age group. The 65–84 age group tended to have shorter duration of regular home visits and more frequent emergency house calls (Supplementary Appendix 4). The AUC of the 3-factor risk score model for the 65–84 age group was 0.766, while for the over 85 age group, it was 0.643.

## Discussion

Using claims data from two Japanese cities, we developed and internally validated a multivariable risk prediction model and scoring system to predict frequent emergency house calls. This risk score showed good discrimination and calibration, and satisfactory internal validity. It provides a useful and easily applicable tool for identifying high-risk patients who may require frequent emergency house calls in the community. The home healthcare team should inform patients and families at high risk for frequent emergency house calls and be prepared to contact their health-care provider easily in the unanticipated events. This risk score may also be used as a trigger to initiate advanced care planning for patients who are at a high risk of having frequent emergency house calls.

Our findings regarding the association between cancer patients and frequent emergency house calls are consistent with a previous study reporting that cancer patients are almost seven times more likely to become frequent attenders at primary care after-hours services compared with non-cancer patients [26]. According to a previous study, cancer in the digestive or respiratory system was the most frequent reason for cancer patients’ use of primary care after-hours services [27]. Another previous study showed that the most common complaints in patients with advanced cancer in the emergency department were pain, shortness of breath, and vomiting, which could also be the reason for emergency house calls [28]. In addition, as “death” is one of the major reasons for emergency house calls in Japan [14, 15], calls due to end-of-life care may be included for cancer patients.

Since cancer patients often experience a rapid decline in physical status, appropriate and timely symptom management and palliative care are necessary to continue their stay at home. Despite offering higher quality end-of-life care compared to the inpatient palliative care units, home palliative care remains uncommon in Japan [29]. Indeed, while more than half of the Japanese people stated that they would prefer to stay at home even when facing their end of life, especially in cancer area [30], most cancer deaths occur in general wards of hospitals (72%), followed by palliative care unit (13%); only 11% of deaths occurred at home in 2016 [31]. To provide end of life care in accordance with the wishes of cancer patients, it is necessary to further establish a system that can handle frequent emergency house calls and provide palliative care at home.

We found that frequent emergency house calls were more likely to occur in patients with high care need levels. This finding may be explained as follows: Higher level of care needed is associated with fever events, and fever is a significant reason for emergency house calls [14]. A previous study in Japan found that fever was more likely in patients with care need levels ≥3 than ≤2, and the conditions most likely to cause fever were pneumonia/bronchitis, skin and soft tissue infections, and urinary tract infections [32]. The authors explained that this was due to an increased risk of aspiration because of decreased strength to cough and increased susceptibility to infections caused by decreased muscle strength and poor nutritional status.

Home oxygen use was associated with frequent emergency house calls. This is consistent with a study in Japan, in which dyspnea was a common chief complaint and there was an association between emergency house calls for dyspnea and home oxygen use [14]. Another study has shown that chronic obstructive pulmonary disease (COPD) is more prevalent among those requiring frequent primary care after-hours services, and that complications and exacerbations of chronic diseases are the reasons for this help-seeking behavior [26].

Our results shows that the AUC is higher for those who are 65–84 years old, making this predictive model more applicable. This may be because the 65–84 age group is dominated by patients with cancer and home oxygen therapy, which are included in the 3-factor risk score, while the over 85 age group tends to have more patients with stable chronic diseases. In addition, the decision to request an emergency house call may be more greatly influenced by caregiver factors in very old patients, making the prediction more difficult.

This risk score would be useful to allocate medical resources and maintain a home medical care system in the community. After the Ministry of Health, Labour, and Welfare introduced HCSCs in 2006, enhanced HCSCs, which required the appointment of three or more full-time doctors, were institutionalized in 2012 [12]. Although the number of HCSCs facilities are increasing, enhanced HCSCs account for only a small percentage of the total HCSCs (approximately 24% in 2018) [13]. Moreover, many general clinics do not meet HCSCs requirements while providing home visits [13]. Most of these clinics are in solo practice and have difficulties providing three or more full-time doctors [33]. Therefore, our tool would be helpful for identifying high-risk patients who may require the frequent emergency house calls and reduce the burden on primary care physicians, especially for solo practitioners, by associating high-risk patients to well-staffed medical institutions, such as enhanced HCSCs.

Our tool is based on information that is readily available in a primary care setting. Therefore, this score can indicate the risk at the start of the regular home visits to allow for targeting a timely approach for high-risk patients. Furthermore, because this score contains only three factors, it is easy to remember and can be quickly calculated in clinical practice.

To the best of our knowledge, this is the first study to develop a risk prediction model for the frequent emergency house calls among older people who receive regular home visits. However, this study has several limitations. First, we did not externally validate the proposed model. Since we derived the study population from two different suburbs of Tokyo, the results may be applicable to other suburbs in large cities in Japan. However, external validation using other cohorts with different regional characteristics would be necessary to confirm the generalizability. Furthermore, to build a prediction model that could be implemented across Japan, future studies using nationwide data are necessary. Second, we did not examine some potential predictors that are known risk-factors, such as the urethral catheter placement [14], because information on these factors was not available. Third, some clinical information generally obtained in clinical settings (such as symptoms, laboratory data, and imaging findings) were unavailable in the database. Fourth, although the instances in which patients and their families perceive the need to request emergency house calls may be influenced by appropriate symptom management, enhanced home medical care, palliative care with team coordination, and family caregiver education and support, we were unable to consider these factors. These factors should be included to improve risk score performance in future studies.

## Conclusions

This easy-to-use risk scoring allows physicians to prospectively identify patients who are at high risk for emergency house calls. It can help reduce the physical and psychological burdens placed on primary care physicians, by taking measures such as clustering high-risk patients in well-equipped medical facilities, ultimately helping to preserve home medical care in the community.

## Supplementary Information

**Additional file 1: Supplementary Appendix 1.** List of medical diagnosis categories and International Classification of Diseases 10th Revision codes.

**Additional file 2: Supplementary Appendix 2.** Distribution of the average number of emergency house calls per month.

**Additional file 3: Supplementary Appendix 3.** Distribution of the total score.

**Additional file 4: Supplementary Appendix 4.** Comparison of patient characteristics across age groups.

## Data Availability

The data that support the findings of this study are available from Tsukuba city and Kashiwa city but restrictions apply to the availability of these data, which were used under license for the current study, and so are not publicly available. Data are however available from the authors upon reasonable request and with permission of Tsukuba city and Kashiwa city.
