# Supplementary material for: Development and validation of a risk score to predict the frequent emergency house calls among older people who receive regular home visits
Source: BMC Prim Care. 2022 May 26;23:132. doi: 10.1186/s12875-022-01742-7 (PMC9137049; doi:10.1186/s12875-022-01742-7)
Supplement: Supplementary file 1 — Additional file 1: Supplementary Appendix 1. List of medical diagnosis categories and International Classification of Diseases 10th Revision codes. [file 12875_2022_1742_MOESM1_ESM.docx]

**Supplementary Appendix1. List of medical diagnosis categories and International Classification of Diseases 10th Revision codes**

| **Medical diagnosis category** | **ICD-10 codes*** |
| --- | --- |
| 1. Cerebrovascular diseases: |  |
| - Hemorrhagic stroke | I60–I62 |
| - Ischemic stroke | I63 |
| - Other cerebrovascular diseases* | I64–I69 |
| 2. Cardiac diseases: |  |
| - Ischemic heart disease | I20–I25 |
| - Arrhythmia | I44, I45, I47–I49 |
| - Heart failure | I50 |
| - Other cardiac diseases | I01, I05–I09, I11, I13, I30–I43, I51, I52 |
| 3. Cancer | C00–C097 |
| 4. Lower respiratory tract diseases |  |
| - COPD | J43, J44 |
| - Pneumonia | J12–18, J69 |
| - Other lower respiratory tract diseases | A15, A16, J20–J22, J40–J42, J45–J47, J60–J68, J70, J80–J86, J90–J94 |
| 5. Joint diseases |  |
| - Rheumatoid arthritis | M05, M06 |
| - Other arthropathies | M00–03, M07, M10–M25 |
| - Dorsopathies (disorders of the back or spine) | M40–M54 |
| 6. Dementia | F00–F03, G30 |
| 7. Parkinson’s disease | G20 |
| 8. Diabetes | E10–E14 |
| 9. Visual or hearing impairment |  |
| - Visual impairment | H53, H54 |
| - Hearing impairment | H90, H91 |
| 10. Fractures |  |
| - Femur fractures | S72 |
| - Other fractures | S02, S12, S22, S32, S42, S52, S62, S82, S92, T02, T08, T10, T12 |

Abbreviations: COPD = chronic obstructive pulmonary disease, ICD-10 = International Classification of Diseases 10th Revision.

*Including unspecified stroke and sequelae of cerebrovascular disease.
