# Supplementary figures and images for: Development and validation of a risk score to predict the frequent emergency house calls among older people who receive regular home visits
Source: BMC Prim Care. 2022 May 26;23:132. doi: 10.1186/s12875-022-01742-7 (PMC9137049; doi:10.1186/s12875-022-01742-7)

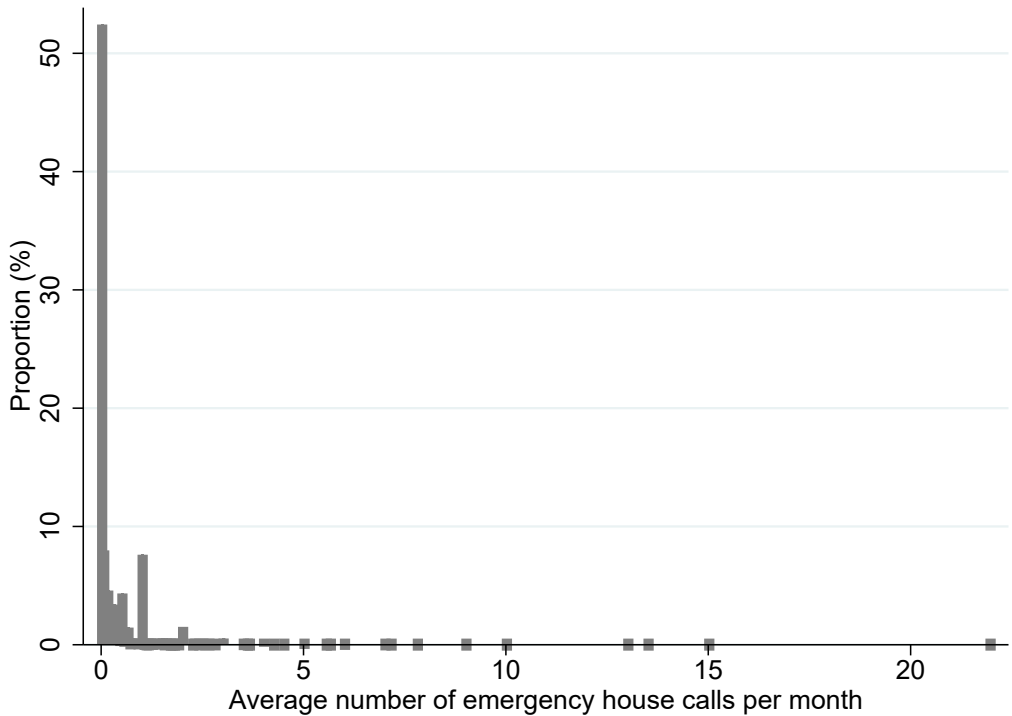

Supplement: Supplementary file 2 — Additional file 2: Supplementary Appendix 2. Distribution of the average number of emergency house calls per month. [file 12875_2022_1742_MOESM2_ESM.pdf]

## Slide 1
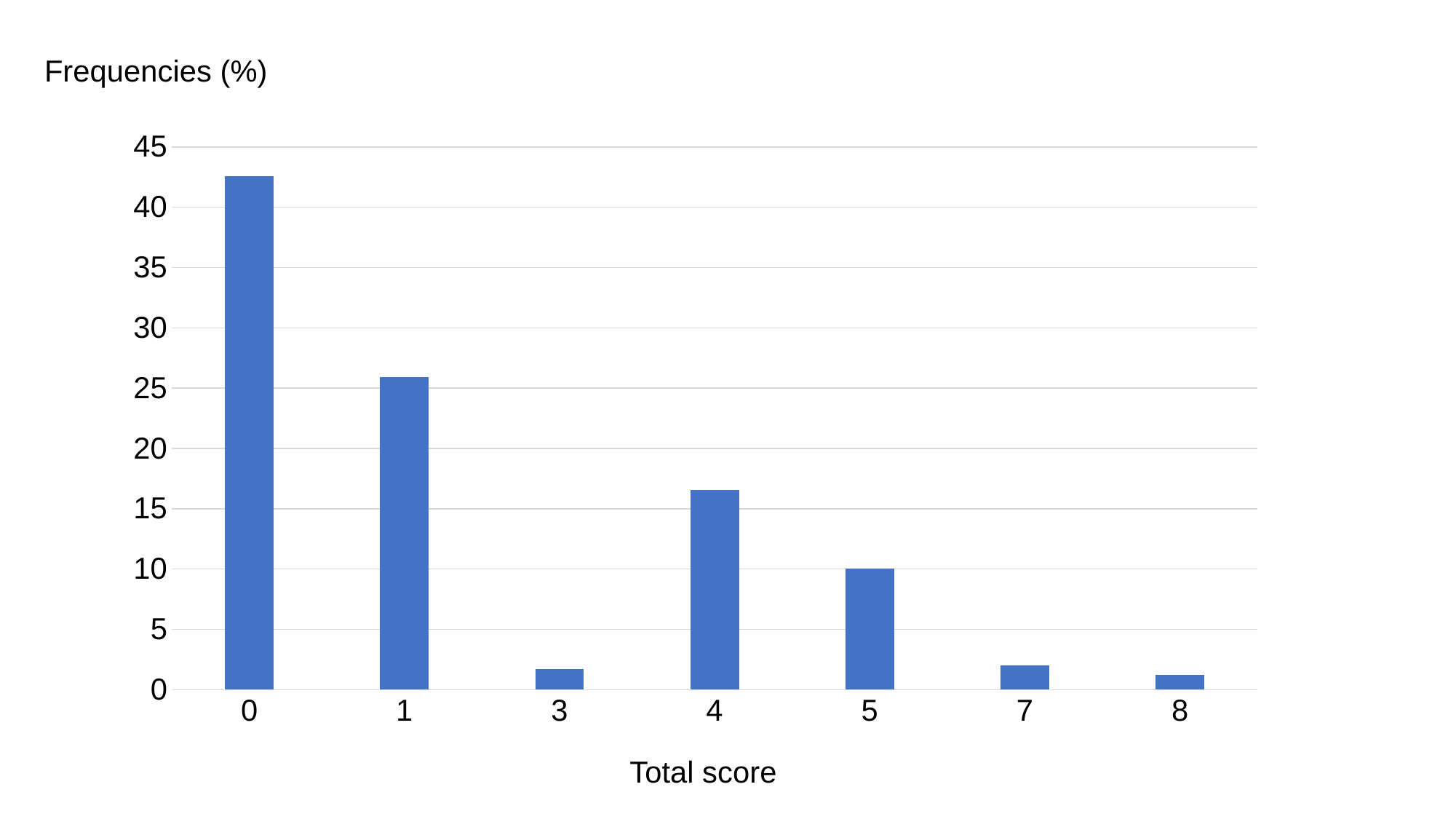

Frequencies (%)
### Chart
| Category | |
|---|---|
| 0 | 42.55 |
| 1 | 25.94 |
| 3 | 1.72 |
| 4 | 16.57 |
| 5 | 10.02 |
| 7 | 1.98 |
| 8 | 1.21 |Total score

Supplement: Supplementary file 3 — Additional file 3: Supplementary Appendix 3. Distribution of the total score. [file 12875_2022_1742_MOESM3_ESM.pptx]
