# Supplementary material for: Development and validation of a risk score to predict the frequent emergency house calls among older people who receive regular home visits
Source: BMC Prim Care. 2022 May 26;23:132. doi: 10.1186/s12875-022-01742-7 (PMC9137049; doi:10.1186/s12875-022-01742-7)
Supplement: Supplementary file 4 — Additional file 4: Supplementary Appendix 4. Comparison of patient characteristics across age groups. [file 12875_2022_1742_MOESM4_ESM.docx]

**Supplementary Appendix 4. Comparison of patient characteristics across age groups.**

|  | 65-84 years group | Over 85 years group |  |
| --- | --- | --- | --- |
|  | N = 2,400 | N = 2,488 |  |
|  | n (%) | n (%) | P value |
| **Mean age, years (SD)** | 78.0 (4.8) | 90.0 (3.9) | <0.001 |
| **Age category (years)** |  |  | <0.001 |
| 65−74 | 505 (21.0) | - |  |
| 75−84 | 1895 (79.0) | - |  |
| 85-94 | - | 2131 (85.7) |  |
| ≥ 95 | - | 357 (14.4) |  |
| **Gender: male** | 1169 (48.7) | 803 (32.3) | <0.001 |
| **Medical procedure at home** |  |  |  |
| Self-injection | 66 (2.8) | 33 (1.3) | <0.001 |
| Central venous nutrition | 44 (1.8) | 20 (0.8) | 0.002 |
| Enteral nutrition | 11 (0.5) | 3 (0.1) | 0.032 |
| Home oxygen therapy | 183 (7.6) | 109 (4.4) | <0.001 |
| Use of ventilator/ tracheostomy performed | 28 (1.2) | 2 (0.1) | <0.001 |
| Urinary self- catheterization | 10 (0.4) | 8 (0.3) | 0.583 |
| **Long-term care need levels** |  |  | 0.065 |
| Care need level 1 | 388 (16.2) | 462 (18.6) |  |
| Care need levels 2–3 | 1069 (44.5) | 1100 (44.2) |  |
| Care need levels 4–5 | 943 (39.3) | 926 (37.2) |  |
| **Medical diagnosis at the start of regular home visit** |  |  |  |
| Cerebrovascular diseases | 960 (40.0) | 993 (39.9) | 0.950 |
| Cardiac disease | 1195 (49.8) | 1588 (63.8) | <0.001 |
| Lower respiratory tract disease | 1082 (45.1) | 1158 (46.5) | 0.306 |
| Joint diseases | 1419 (59.1) | 1559 (62.7) | 0.011 |
| Dementia | 916 (38.2) | 1195 (48.0) | <0.001 |
| Parkinson’s disease | 247 (10.3) | 88 (3.5) | <0.001 |
| Diabetes | 868 (36.2) | 747 (30.0) | <0.001 |
| Vision or hearing impairment | 164 (6.8) | 178 (7.2) | 0.660 |
| Fractures | 372 (15.5) | 520 (20.9) | <0.001 |
| Cancer | 856 (35.7) | 548 (22.0) | <0.001 |
| **Month of receiving regular home visits: median (IQR)** | 6 (2–12) | 8 (3–13) | <0.001 |
| **Frequent emergency house calls*** | 337 (14.0) | 297 (11.9) | 0.029 |

Abbreviations: SD = standard deviation, IQR = inter quartile range.

*Emergency house calls once per month or more, on average, during each observation period.
